# Multilevel multinomial regression analysis of factors associated with birth weight in sub-Saharan Africa

**DOI:** 10.1038/s41598-024-58517-6

**Published:** 2024-04-22

**Authors:** Meklit Melaku Bezie, Getayeneh Antehunegn Tesema, Beminate Lemma Seifu

**Affiliations:** 1 Department of Public Health, Institute of Public Health, College of Medicine and Health Sciences and Comprehensive Specialized Hospital, University of Gondar, Gondar, Ethiopia; 2 Department of Epidemiology and Biostatistics, Institute of Public Health, College of Medicine and Health Sciences and comprehensive specialized hospital, University of Gondar, Gondar, Ethiopia; 3 Department of Public Health, College of Medicine and Health Sciences, Samara University, Samara, Ethiopia

**Keywords:** Birth weight, Sub-Saharan Africa, Multilevel multinomial logistic regression, Health care, Medical research, Risk factors

## Abstract

Birth weight significantly determines newborns immediate and future health. Globally, the incidence of both low birth weight (LBW) and macrosomia have increased dramatically including sub-Saharan African (SSA) countries. However, there is limited study on the magnitude and associated factors of birth weight in SSA. Thus, thus study investigated factors associated factors of birth weight in SSA using multilevel multinomial logistic regression analysis. The latest demographic and health survey (DHS) data of 36 sub-Saharan African (SSA) countries was used for this study. A total of a weighted sample of 207,548 live births for whom birth weight data were available were used. Multilevel multinomial logistic regression model was fitted to identify factors associated with birth weight. Variables with p-value < 0.2 in the bivariable analysis were considered for the multivariable analysis. In the multivariable multilevel multinomial logistic regression analysis, the adjusted Relative Risk Ratio (aRRR) with the 95% confidence interval (CI) was reported to declare the statistical significance and strength of association. The prevalence of LBW and macrosomia in SSA were 10.44% (95% CI 10.31%, 10.57%) and 8.33% (95% CI 8.21%, 8.45%), respectively. Maternal education level, household wealth status, age, and the number of pregnancies were among the individual-level variables associated with both LBW and macrosomia in the final multilevel multinomial logistic regression analysis. The community-level factors that had a significant association with both macrosomia and LBW were the place of residence and the sub-Saharan African region. The study found a significant association between LBW and distance to the health facility, while macrosomia had a significant association with parity, marital status, and desired pregnancy. In SSA, macrosomia and LBW were found to be major public health issues. Maternal education, household wealth status, age, place of residence, number of pregnancies, distance to the health facility, and parity were found to be significant factors of LBW and macrosomia in this study. Reducing the double burden (low birth weight and macrosomia) and its related short- and long-term effects, therefore, calls for improving mothers' socioeconomic status and expanding access to and availability of health care.

## Introduction

The birth weight of a baby is a critical indicator of their health in the short and long term^1,2^. An estimated 38% of all under-five deaths globally were reported to have occurred in Sub-Saharan Africa (SSA)^3^. Macrosomia affects 3–15% of pregnancies worldwide, with high-income countries having the highest percentage (5–20%)^4,5^. An estimated 20 million (14.6%) newborns worldwide suffer from low birth weight^6^.

According to a global consensus, a low birth weight (LBW) is defined as a baby's weight of less than 2500 g at birth and macrosomia if the birth weight greater than 4000 g, ideally measured in the first hour of life^7^. Given that they are more susceptible to dying than heavier ones, it is one of the main causes of neonatal, infant, and childhood mortality and morbidity^7,8^. In sub-Saharan Africa, the incidence of low birth weight has risen from 4.4 million in 2000 to 5 million in 2015^9^.

Both macrosomia and low birth weight are strongly linked with early childhood mortality and future risks of chronic morbidities^10–12^. Long-term health consequences like impaired motor function, poor cognitive development, and an increased risk of chronic illnesses like diabetes, cancer, and cardiovascular diseases are all closely associated with them^13–15^. In addition to neonatal and infant mortality, low birth weight has a substantial impact on physical and developmental health issues in subsequent childhood and adulthood^16,17^. It causes stunted growth in children and a higher prevalence of chronic illnesses in adults, including cancer, diabetes mellitus, hypertension, and cardiovascular disease^18,19^.

Moreover, it has long-term effects like impaired cognitive function and poor academic achievement^20–22^. Low birth weight is generally used as a summary indicator of multilayered public health issues like poor utilisation of pregnancy-related health services, illness, and malnutrition in mothers.

Previous studies have found that advanced maternal age^23–25^, multiple pregnancies^26,27^, multiparity^28,29^, obstetric complications^30,31^, underlying maternal chronic conditions (i.e. hypertensive disorder of pregnancy, gestational diabetes)^32,33^, infections (such as malaria, HIV)^34,35^, maternal nutritional status^36,37^ and substance use^38^ were found significant determinants of birth weight. The highest prevalence of LBW and macrosomia can demonstrate poor maternal health status, maternal malnutrition (undernutrition and overnutrition), inappropriate pregnancy care, and deprived socio-economic status of mothers^39–41^.

According to previous studies, macrosomia was linked to chronic conditions like diabetes, heart disease, and obesity^42,43^. Research has shown that LBW, preterm, early neonatal death, and infant and under-five mortality are all associated with compromised maternal reproductive health^44,45^. Similarly, poor maternal health like obesity, underlying medical conditions (e.g. Diabetic mellitus, hypertension, cardiovascular disease), and substance use like smoking will also lead to increased risk of macrosomia^42,46^.

Hence, both macrosomia and LBW have long-term effects that place a significant financial strain on Sub-Saharan Africa (SSA) unless public health initiatives are made to address the major risk factors associated with them. Consequently, in order to develop efficient preventive measures to lower the incidence of LBW and macrosomia, underlying factors should be identified. In order avoid information loss and obtain a reliable estimate, we therefore used the multilevel multinomial logistic regression model. The present study employed a methodology that utilised the pooled DHS data of 36 sub-Saharan African countries, resulting in a substantial sample size. This could potentially enhance the study's external validity and power. A comprehensive view of SSA can be obtained by utilising a multilevel approach that takes the neighbourhood effect into account. Furthermore, birth weight has been categorised as a binary outcome in earlier research by being assigned the labels LBW/normal. But as you can see, there is a loss of information because macrosomia is a problem that might not be similar to normal birth weight, so treating macrosomia and normal birth weight as normal is not statistically appropriate.

## Methods

### Data source and sampling procedure

This study was a community based cross-sectional study based on the Demographic and Health Survey (DHS) data of 36 sub-Saharan African countries. To obtain the samples, the DHS consistently employed a multi-stage sampling technique for each country. The primary sampling unit and secondary sampling unit were Enumeration Areas (EAs) and households, respectively. This study made use of the Kids Record dataset (KR file). This survey's details, such as its design, questionnaires, and sampling methods, have been publicly released^47^. Table 1 presents the weighted sample size for each country (Table 1).

**Table 1:** Sample size in each country, and total sample size in sub-Saharan Africa.

Sub-Saharan African region	Country	Total weighted frequency	Percentage (%)
East Africa	Kenya	6146	2.96
	Ethiopia	1502	0.72
	Comoros	2171	1.05
	Rwanda	7383	3.56
	Uganda	10,266	4.95
	Madagascar	5070	2.44
	Mozambique	5998	2.89
	Malawi	14,600	7.03
	Tanzania	6386	3.08
	Burundi	10,922.97	5.26
	Zambia	7897	3.80
	Zimbabwe	5274	2.54
Southern Africa	Lesotho	2595	1.25
	Namibia	4100	1.98
	Swaziland	2368	1.14
	South Africa	3158	1.52
West Africa	Burkina Faso	9779	4.71
	Benin	8235	3.97
	Cote di’ viore	4511	2.17
	Ghana	3434	1.65
	Gambia	4689	2.26
	Guinea	3859	1.86
	Mali	3701	1.78
	Nigeria	8093	3.90
	Niger	3091	1.49
	Serra Leone	5811	2.80
	Senegal	6964	3.36
	Togo	4008	1.93
	Liberia	1510	0.73
Central Africa	Angola	7377	3.55
	Democratic Congo	7409	3.57
	Congo	13,922	6.71
	Cameroon	6921	3.33
	Gabon	4646	2.24
	Sao Tome	1505	0.73
	Chad	2244	1.08

### Measurement of variables

The study's outcome variable was birth weight, which was classified as low, normal, and macrosomia. We included live births for whom birth weights were recorded. Maternal education status, household wealth status, age, media exposure, sex of the head of the household, women's autonomy in making health care decisions, marital status, wanted child, child's sex, number of pregnancies, parity, distance to health facility, duration of birth interval, number of ANC visits, sub-Saharan African region and residence were the independent variables considered in the study (Table 2).

**Table 2:** List of study variables.

Study variables	Description and categories
Outcome variable	Weight of the child at birth in grams, categorized as normal birth weight = 0 “2500–4000 g”, low birth weight = 1 “ < 2500 g” and macrosomia = 2 “ > 4000 g”
Independent variables	
Residence	Type of place of residence 1 = urban 2 = rural
Maternal age	Maternal age during childbirth (0 = 15–24 years, 1 = 25–34 years and 2 = 35–49 years)
Sex of child	Sex of child (0 = female and 1 = male)
Women health care decision making autonomy	Person who usually decides on visits to family or relatives (0 = respondent alone, 1 = jointly with husband or partner and 2 = husband or partner or relative alone)
Maternal education	Education level of mother (0 = no formal education, 1 = primary, 2 = secondary and 3 = higher)
Household wealth status	Household wealth quintile (0 = poorest, 1 = poorer, 2 = middle, 3 = richer and 4 = richest)
Maternal occupation	Working status of the mother (0 = not working and 1 = working)
Media exposure	Media exposure of the mother (0 = have no exposure to all of reading newspaper, listening radio and watching television and 1 = had exposure to either of reading newspaper, listening radio or watching television)
Sex of household head	Sex of household head (0 = male and 1 = female)
Distance to health facility	Perceived distance to reach the health facility (0 = not a big problem and 1 = a big problem)
Marital status	Current marital status of the mother (0 = not married, 1 = married and 2 = divorced/widowed/separated)
Parity	Number of children ever born (0 = one, 1 = two–three and 2 = four and above)
Number of ANC visits	Number of ANC visit for the recent pregnancy (0 = no, 1 = one-three visits and 2 = four and above visits)
Sub-Saharan Africa region	Sub-Saharan Africa region (0 = East Africa, 1 = Southern Africa, 2 = Central Africa and 3 = West Africa)
Duration of birth interval	Duration of preceding birth interval (0 = less than 24 months, 1 = 24–59 months and 2 = 60 months and above)
Number of pregnancy	Number of pregnancy (0 = single and 1 = multiple)

### Data management and analysis

All the analysis was based on the weighted data. Data management and analysis were done using STATA-17 software. The outcome variable (birth weight) has three categories; LBW, normal and macrosomia.

A multilevel multinomial logistic regression model was fitted to examine the association between individual and community-level variables with macrosomia and LBW, using normal birth weight groups as a reference category. Compared to the standard multinomial logistic regression model, the multilevel multinomial logistic regression analysis has advantages. It reduces parameter overestimation and obtain more accurate estimates of the model parameters because the DHS survey is hierarchical. To estimate the variation between clusters, we used clusters/EAs as a random variable. Furthermore, multilevel modelling can estimate cluster-level effects, also known as random effects, concurrently with measures of associations of community-level variables, such as residence, and region of sub-Saharan Africa. Additionally, birth weight was treated as a binary outcome in previous studies on factors related to birth weight (LBW vs normal)^48,49^. While birth weight has a multinomial nature (low birth weight, normal, and macrosomia). Therefore, treating birth weight as binary in nature results in a loss of information and is not informative scientifically and not biologically plausible. Given the above-mentioned rationales, multilevel multinomial modeling was fitted. Considering the nature of outcome variable, we fitted both multilevel binary logistic regression and multilevel multinomial logistic regression models by treating birth weight as binary and multiple categories, respectively. Given the analysis results obtained from the these regressions, we choose multilevel multinomial logistic regression model (Supplementary File 1).

Using a multinomial family and logit link, Generalised Structural Equation Modelling (GSEM) was used to implement the multilevel multinomial logistic regression analysis. For the multilevel multinomial logistic regression analysis, four models were built. To find out how much cluster variation there was in the birth weight categories, the first model was an empty one with no explanatory variables. Individual-level variables were used to adjust the second model, community-level variables were used to adjust the third model, and both individual- and community-level variables were fitted simultaneously to the fourth model. The model with the smallest deviance was selected.

The percentage of the total observed individual variation in low birth weight and macrosomia that can be attributed to cluster variations is measured by the intra-class correlation coefficient (ICC), which measures the degree of heterogeneity of birth weight categories between clusters. ICC= ${\partial }^{2}/({\partial }^{2}+\frac{{\pi }^{2}}{3}$^50^, where;

$\partial$^2^ indicates that cluster variance.

In the multilevel model, PCV quantifies the overall variation attributable to both individual- and community-level factors in contrast to the null model.

$$ {\text{PCV}} = \, \frac{{{\text{var }}\left( {\text{null model}} \right) \, - {\text{ var full model}}))}}{{{\text{Var }}\left( {\text{null model}} \right)}}, $$

In the bivariable analysis, variables with p-value < 0.2 were chosen and considered for the multivariable analysis. In the final model, the Adjusted Relative Risk Ratio (aRRR) with a 95% Confidence Interval (CI) was reported to define the significance of the association.

### Ethical consideration

In the case of this study, we have been granted an authorized letter from the measure DHS program for the use the data. DHS is publicly available de-identified data; ethical approval is not needed.

## Results

A total of 207,548 live births with birth weight measurements were included in this study. Of them, 121,192 (58.39%) were from rural areas. More than one-fourth (26.29%) of the mothers had no formal education. About 15.59% and 18.21% of the mothers belonged to the poorest and poorest household quintiles, respectively. The majority (66.04%) of the mothers claimed that perceived distance to the health facility was a big problem. Regarding the number of ANC visits, about 100,616 (48.48%) had 4 ANC visits and above (Table 3).

**Table 3:** Descriptive characteristics of the study participants in Sub-Saharan Africa.

Characteristics	Frequency (n = 206,528)	Percentage (100%)
Residence		
Urban	86,355	41.61
Rural	121,192	58.39
Household wealth status		
Poorest	32,353	15.59
Poorer	37,785	18.21
Middle	41,019	19.76
Richer	46,556	22.43
Richest	49,834	24.01
Maternal educational status		
No education	54,565	26.29
Primary	77,550	37.36
Secondary	65,476	31.55
Higher	9956	4.80
Media exposure		
No	53,024	25.55
Yes	154,523	74.45
Maternal age (in years)		
15–24	61,418	29.59
25–34	100,986	48.66
35–49	45,144	21.75
Maternal working status		
Not working	71,503	34.45
Working	136,044	65.55
Marital status		
Not married	16,378	7.89
Married	175,444	84.53
Divorced/widowed/separated	15,725	7.58
Parity		
1	37,379	18.01
2–3	83,220	40.10
> 3	86,949	41.89
Number of ANC visits		
No	58,141	28.01
1–3	48,790	23.51
≥ 4	100,616	48.48
Duration of birth interval		
< 2 years	25,094	16.33
2–5 years	102,376	66.64
> 5 years	26,154	17.02
Types of pregnancy		
Single	19,980	96.27
Multiple	7747	3.73
Sex of child		
Male	105,226	50.70
Female	102,321	49.30
Distance to health facility		
Not a big problem	137,071	66.04
Big problem	70,477	33.96
Sex of household head		
Male	159,883	Male
Female	47,664	Female
Women autonomy in health care decision making		
Respondent alone	32,020	15.43
Jointly with partners/husband	71,841	34.61
Husband/partner alone	103,686	49.96
Wanted child		
Not wanted	16,116	7.76
Wanted	191,431	92.24
Sex of household head		
Male	159,883	77.03
Female	47,664	22.97
Sub-Saharan African region		
East Africa	83,616	40.29
Southern Africa	12,221	5.89
Central Africa	44,024	21.21
West Africa	67,687	32.61

The prevalence of LBW and macrosomia in sub-Saharan Africa were 10.44% (95% CI 10.31%, 10.57%) and 8.33% (95% CI 8.21%, 8.45%), respectively. The prevalence has varied by country, with LBW prevalence ranging from 6.30% in Rwanda to 16.21% in Comoros and macrosomia prevalence ranging from 1.73% in Chad to 26.68% in Burkina Faso.

### Multilevel multinomial regression analysis results

The ICC indicated that a clustering effect existed, which should be addressed with advanced statistical models such as multilevel modelling to obtain an unbiased standard error and draw meaningful conclusions. The null model's ICC value was 11%, meaning that 89% of the variation in birth weight was attributable to individual variability and that only 11% was caused by cluster variability. Additionally, the final model's PCV value of 0.97 indicated that it explained approximately 97% of the variation in birth weight. Then four models were fitted and compared using LLR and deviance as they were nested. The final model (a model with individual and community-level characteristics) was the best-fitted model for the data since it had the lowest deviance value (Table 4).

**Table 4:** Random effect results.

Parameters	Null model	Model 1	Model 2	Model 3
Community level variance	0.41	0.11	0.17	0.01
ICC	0.11	0.03	0.05	0.003
PCV	ref	0.73	0.59	0.97
LLR	− 127,182.6	− 123,009.7	− 126,259.9	− 122,335.7
Deviance	254,365.2	246,018.4	252,519.8	244,671.4

To identify factors associated with birth weight i.e. low birth weight and macrosomia, a multilevel multinomial logistic regression analysis was fitted. Considering the nature of the DHS data, both individual and community-level variables were considered as independent variables in the model.

Maternal educational status, household wealth status, parity, women's health care decision-making autonomy, sex of household head, marital status, media exposure, maternal age, occupational status, distance to the health facility, sub-Saharan African region, residence, and number of pregnancies had p-value < 0.2 in the bivariable multilevel multinomial regression analysis and considered for the multivariable multilevel multinomial logistic regression analysis. In the multivariable analysis; maternal educational status, household wealth status, maternal age, parity, number of pregnancies, distance to the health facility, residence, and sub-Saharan African region were significantly associated with low birth weight. Mothers who attained primary education, secondary education, and higher had 10% [RRR = 0.90, 95% CI 0.86, 0.93], 21% [aRRR = 0.79, 0.75, 0.83], and 31% [aRRR = 0.69, 95% CI 0.63, 0.76] lower risk of delivering a low birth weight baby compared to mothers who had no formal education, respectively. The risk of having a low birth weight baby decreases with the higher wealth index; poorer [aRRR = 0.94, 95% CI 0.89, 0.98], middle [aRRR = 0.89, 95% CI 0.85, 0.94], richer [aRRR = 0.85, 95% CI 0.81, 0.89] and richest [aRRR = 0.76, 95% CI 0.71, 0.80] had significant reductions. The risks of having low birth weight baby among respondents aged 25–34 and 35–49 years were decreased by 19% [aRRR = 0.81, 95%CI 0.77, 0.84] and 15% [aRRR = 0.85, 95% CI 0.81, 0.90] compared to mothers aged 15–24 years, respectively. Being multiparous was significantly associated with a decreased risk of delivering a low birth weight baby than primiparous mothers. Regarding the number of pregnancies, mothers who had multiple pregnancies were 8.03 times [aRRR = 8.03, 95% CI 7.64, 8.44] a higher risk of having a low birth weight baby than mothers who had single pregnancy. Being a rural resident increased the risk of delivering a low birth weight baby by 1.14 times [aRRR = 1.14, 95% CI 1.09, 1.18] than their counterparts. The risk of giving a low birth weight baby among women who perceived distance to a health facility as a big problem was 1.06 times [aRRR = 1.06, 95% CI 1.03, 1.10] higher compared to those who perceived it as not a big problem. Compared with the East African region, respondents living in Southern Africa [aRRR = 1.14, 95% CI 1.06, 1.22], and West African regions [aRRR = 1.06, 95% CI 1.01, 1.17] were more likely to have children with low birth weight (Table 5).

**Table 5:** Multilevel multinomial regression analysis of factors associated with birth weight (for both low birth weight and macrosomia) in sub-Saharan Africa.

Characteristics	Null model	Model I		Model II		Model III	
		Individual level variables		Community level variables		Both individual and community level characteristics	
		LBW (RRR with 95% CI)	Macrosomia (RRR with 95% CI )	LBW (RRR with 95% CI)	Macrosomia (RRR with 95% CI)	LBW (RRR with 95% CI)	Macrosomia ( with 95% CI)
Maternal educational status							
No		1	1			1	1
Primary		0.87 [0.84, 0.90]	1.45 [1.39, 1.51]			0.90 [0.86, 0.93]*	1.25 [1.20, 1.31]*
Secondary		0.77 [0.74, 0.81]	1.36 [1.30, 1.43]			0.79 [0.75, 0.83]*	1.11 [1.06, 1.17]*
Higher		0.69 [0.63, 0.75]	1.31 [1.19, 1.44]			0.69 [0.63, 0.76]*	1.15 [1.04, 1.26]*
Household wealth status							
Poorest		1	1			1	1
Poorer		0.95 [0.90, 0.99]	1.05 [0.99, 1.11]			0.94 [0.89, 0.98]*	1.06 [1.01, 1.12]**
Middle		0.92 [0.87, 0.96]	1.01 [0.96, 1.06]			0.89 [0.85, 0.94]**	1.05 [0.99, 1.10]
Richer		0.90 [0.86, 0.94]	0.96 [0.91, 1.01]			0.85 [0.81, 0.89]*	1.04 [0.98, 1.10]
Richest		0.83 [0.79, 0.88]	0.99 [0.94, 1.05]			0.76 [0.71, 0.80]*	1.14 [1.07, 1.21]**
Maternal age (in years)							
15–24		1	1			1	1
25–34		0.82 [0.79, 0.85]	0.91 [0.87, 0.96]			0.81 [0.77, 0.84]*	0.97 [0.92, 1.02]
35–49		0.87 [0.82, 0.92]	0.86 [0.82, 0.92]			0.85 [0.81, 0.90]*	0.93 [0.87, 0.98]*
Media exposure							
No		1	1			1	1
Yes		0.99 [0.96, 1.02]	0.92 [0.89, 0.96]			0.97 [0.93, 1.01]	0.99 [0.95, 1.03]
Maternal occupation status							
Not working		1	1			1	1
Working		0.90 [0.87, 0.92]	1.14 [1.10, 1.18]			0.91 [0.88, 1.02]	1.10 [1.06, 1.13]
Marital status							
Not married		1	1			1	1
Currently married		0.92 [0.87, 0.97]	1.20 [1.11, 1.29]			0.94 [0.89, 1.01]	1.10 [1.02, 1.19]*
Divorced/widowed/separated		1.03 [0.96, 1.10]	1.44 [1.32, 1.56]			1.06 [0.99, 1.14]	1.33 [1.23, 1.45]*
Parity							
1		1	1			1	1
2–3		0.84 [0.80, 0.88]	1.31 [1.24, 1.38]			0.84 [0.80, 0.88]*	1.26 [1.20, 1.3]*
≥ 4		0.71 [0.67, 0.74]	1.74 [1.63, 1.85]			0.72 [0.68, 0.76]*	1.54 [1.44, 1.64]*
Number of pregnancies							
Single		1	1			1	1
Multiple		8.05 [7.62, 8.45]	0.48 [0.42, 0.54]			8.03 [7.64, 8.44]**	0.47 [0.41, 0.54]**
Women health care decision making autonomy							
Respondent alone		1	1			1	1
Jointly with partners/husband		0.87 [0.83, 0.91]	0.98 [0.94, 1.03]			0.88 [0.74, 1.04]	0.96 [0.91, 1.01]
Husband/partner alone		1.04 [0.99, 1.09]	1.10 [1.05, 1.16]			1.04 [0.99, 1.09]	1.01 [0.96, 1.07]
Wanted birth							
Not wanted		1	1			1	1
Wanted		0.96[0.91, 1.02]	0.84 [0.80, 0.89]			0.98 [0.92, 1.03]	0.80 [0.75, 0.84]*
Sex of household head							
Male		1	1			1	1
Female		0.99 [0.96, 1.04]	1.00 [0.96, 1.04]			0.99 [0.95, 1.02]	1.02 [0.98, 1.06]
Distance to HF							
Not a big problem		1	1			1	1
A big problem		1.09 [1.05, 1.12]	0.95 [0.91, 1.01]			1.06 [1.03, 1.10]*	0.98 [0.95, 1.02]
Place of residence							
Urban				1	1	1	1
Rural				1.01 [0.98, 1.04]	1.14 [1.10, 1.18]	1.14 [1.09, 1.18]*	0.86 [0.83, 0.97]*
Sub-Saharan Africa region							
East Africa				1	1	1	1
Southern Africa				1.19 [1.12, 1.04]	0.63 [0.58, 0.68]	1.14 [1.06, 1.22]*	0.69 [0.63, 0.76]*
Central Africa				0.99 [0.95, 1.03]	1.77 [1.70, 1.83]	0.92 [0.88, 1.01]	1.77 [1.70, 1.84]*
West Africa				1.15 [1.11, 1.19]	0.82 [0.78, 0.85]	1.06 [1.01, 1.17]*	0.89 [0.85, 0.93]*
Constant	− 2.045415	− 1.432290	− 2.88724	− 2.139871	− 2.403731	− 1.372618	− 2.798989

*p-value < 0.05, **p-value < 0.01. **aRRR adjusted relative risk ratio, *CI* confidence interval.

In the final multilevel multinomial logistic regression analysis; maternal educational status, household wealth status, maternal age, parity, number of pregnancies, marital status, wanted pregnancy, residence, and sub-Saharan African region were significantly associated with macrosomia. Maternal level of education has a significant association with macrosomia; mothers who attained primary education, secondary education, and higher education were 1.25 [aRRR = 1.25, 95% CI 1.20, 1.31], 1.11 times [aRRR = 1.11, 95% CI 1.06, 1.17] and 1.15 times [aRRR = 1.15, 95% CI 1.04, 1.26] times higher risk of having a macrosomic baby than those who didn't attain formal education, respectively. Mothers in the poorer household wealth [aRRR = 1.06, 95% CI 1.01, 1.12] and richest household wealth status [aRRR = 1.14, 95% CI 1.07, 1.21] had an increased risk of delivering a macrosomic baby compared to those in the poorest households. The risk of having a macrosomic baby among mothers aged 35–49 years was decreased by 7% [aRRR = 0.93, 95% CI 0.87, 0.98] than those aged 15–24 years. Babies born to married and divorced/widowed/separated mothers had 1.10 times [aRRR = 1.10, 95% CI 1.02, 1.19] and 1.33 times [aRRR = 1.33, 95% CI 1.23, 1.45] higher risk of macrosomia compared to unmarried women, respectively. Regarding parity and number of pregnancies, the risk of having a macrosomic baby increased as parity increased, and mothers with multiple pregnancies had a lower risk of giving a macrosomic baby [aRRR = 0.47, 95% CI 0.41, 0.54] compared to the singletons. Being rural decreased the risk of macrosomia by 14% [aRRR = 0.86, 95% CI 0.83, 0.97] compared to urban. Compared to the East African region, mothers living in southern Africa and west African regions had a lower risk of delivering a macrosomic baby while those in the Central African region had a higher risk of macrosomia (Table 5).

## Discussion

In this study, we investigated into the birth weight-related factors in sub-Saharan Africa, specifically low birth weight and macrosomia. Birth weight was significantly correlated with the following factors: maternal education, household wealth status, maternal age, parity, number of pregnancies, residence, wanted birth, and sub-Saharan Africa region.

A significant association was found between low birth weight and macrosomia and the mother's place of residence. Mothers living in a rural area had a higher risk of delivering low birth weight babies in contrast they were at lower risk of giving a macrosomic baby. This was consistent with studies reported in Developing countries^51^, Bangladesh^52^, India^53^, and the United States of America^54^. This might be because reproductive health care services in SSA are highly skewed in urban areas, and therefore rural pregnant mothers have poor access to these health care services, health information related to pregnancy, and nutritional awareness^55,56^. In addition, rural resident pregnant mothers are more susceptible to psychosocial stress, which in turn increases the release of cortisol, and catecholamine, which is linked with low birth weight^57,58^. The risk of giving low birth weight babies was lower among educated mothers than those who didn't have formal education while the risk of macrosomia was higher among educated than those who didn't have formal education. This is consistent with findings reported in Malawi^28^, Brazil^59^, and Eastern Nepal^60^.

Similarly, the risk of having a low birth weight baby was decreased, and the risk of having a macrosomic baby was increased as the household wealth status increased. It was supported by evidence reported in China^61,62^, and Ethiopia^63^. This could be due to pregnant mothers who are less educated are commonly have poor socio-economic status, which in turn results in poor maternal diet which is responsible for low birth weight^64,65^. In contrast, those who are educated are aware of maternal nutrition like diversified food which is a feature of good household wealth, this might cause excessive pregnancy weight gain and is responsible for increased fetal size^62^. The lower level of education has also been linked with corresponding limited access to maternal health care^66^. We speculated that educated women are more likely to adhere to health messages either because of the cognitive priming that education affords. Another important predictor of low birth weight and macrosomia was multiple pregnancies. It was consistent with study findings in Korea^67^. This could be because multiple pregnancies are identified as high-risk pregnancies, closely linked with a higher risk of maternal and fetal morbidity and mortality^68^.

Studies showed that multiple pregnancies are at increased risk of preterm birth, congenital anomalies, and twin-twin transfusion syndrome^1^. Additionally, multiparity was found to be associated with a lower risk of low birth weight and a higher risk of macrosomia. This was in line with many previous researches^69–71^, the possible reason is that multiparous mothers have experience in improving pregnancy outcomes and adhering to pregnancy care. Moreover, advanced maternal age was significantly associated with a lower risk of low birth weight and macrosomia. This was supported by previous studies^25,72^, it could be due to the increased risk of chronic medical conditions like hypertension, and diabetes as well as nutritional depletion could be responsible for the increased risk of low birth weight and macrosomia^73^.

Another significant predictor was pregnancy wantedness, which was consistent with studies reported in Ecuador^74^ and Colombia^75^. This could be because mothers with wanted pregnancies have more adhered to maternal health care services like antenatal care and nutritional supplementations^76^. A woman who perceives distance to a health facility as a big problem has a higher risk of delivering a low birth weight baby. It was consistent with study findings in China^77^, Thailand^78^, and India^79^. This could be due to the reason that the healthcare access problem is the main factor for adverse birth outcomes like low birth weight, it highlights that there is a need to make maternal healthcare services available and accessible to the community^80^. This study has both strengths and limitations. The present study employed a methodology that utilised the pooled DHS data of 36 sub-Saharan African countries, resulting in a substantial sample size. This could potentially enhance the study's external validity and power. A comprehensive view of SSA can be obtained by utilising a multilevel approach that takes the neighbourhood effect into account. Furthermore, birth weight has been categorised as a binary outcome in earlier research by being assigned the labels LBW/normal. But as you can see, there is a loss of information because macrosomia is a problem that might not be similar to normal birth weight, so treating macrosomia and normal birth weight as normal is not statistically appropriate. Despite the above strengths, the DHS data is cross-sectional, and as such causal relationships cannot be made. Because the retrospective data on their prior history was gathered, it is therefore vulnerable to recall bias. Furthermore, as we conducted a secondary data analysis important variable like maternal medical conditions were not available.

## Conclusion

In this study, low birth weight and macrosomia were major public health problems in SSA. We identified several factors associated with low birth weight and macrosomia. Higher level of education, improved wealth, multiparity, multiple pregnancies, perceived distance to a health facility as a big problem, and being a rural resident was significantly associated with low birth weight. Similarly, a higher level of education, improved wealth, multiparity, multiple pregnancies, advanced maternal age, wanted pregnancy, maternal age, and being a rural resident were significant predictors of macrosomia. Therefore, MNCH programs in SSA should target high risk groups the prevention of low birth weight and macrosomia.

### Supplementary Information

Supplementary Information.

## Data Availability

The datasets generated and/or analysed during the current study are available in the https://dhsprogram.com/data/dataset_admin/login_main.cfm.

## Abbreviations

aRRR: Adjusted relative risk ratio
DHS: Demographic health survey
CI: Confidence interval
EAs: Enumeration areas
ICC: Intra-cluster correlation coefficient
LLR: Log likelihood ratio
SSA: Sub-Saharan Africa
