# Supplementary material for: Multilevel multinomial regression analysis of factors associated with birth weight in sub-Saharan Africa
Source: Sci Rep. 2024 Apr 22;14:9210. doi: 10.1038/s41598-024-58517-6 (PMC11035683; doi:10.1038/s41598-024-58517-6)
Supplement: Supplementary file 1 — Supplementary Information. [file 41598_2024_58517_MOESM1_ESM.docx]

**Statistical analysis**

Depending on the nature of the outcome variable we fitted multilevel binary logistic regression and multilevel multinomial logistic regression analysis models. Due to the relative simplicity, wide availability of codes and speed of model convergence, previous studies treated birth weight as binary outcome such as LBW vs normal or Macrosomia vs normal. However, this could cause of loss of information. Therefore, we fitted the multilevel multinomial logistic regression analysis in the GSEM framework.

We fitted both models and checked the presence of difference in statistical significance of predictors and effect measure size. As you can see on Table 1 and Table 2, there is difference in estimates and significance of association for some of the variables (Table 1 and Table 2). For the main study, given the statistical implication and power of the study, we reported the multilevel multinomial logistic regression analysis results.

Table 1: Multivariable multilevel binary logistic regression analysis of LBW in SSA

| Variable | Category | LBW (Adjusted Odds Ratio (AOR) with 95% Confidence Interval (CI)) |
| --- | --- | --- |
| Maternal educational status | No | 1 |
|  | Primary | 0.89 [0.85, 0.92]^*^ |
|  | Secondary | 0.82 [0.79, 0.86]^*^ |
|  | Higher | 0.76 [0.69, 0.83]^*^ |
| Household wealth status | Poorest | 1 |
|  | Poorer | 0.94 [0.89, 0.98] ^*^ |
|  | Middle | 0.89 [0.85, 0.93] ^*^ |
|  | Richer | 0.86 [0.82, 0.90] ^*^ |
|  | Richest | 0.76 [0.72, 0.80] ^*^ |
| Maternal age (in years) | 15-24 | 1 |
|  | 25-34 | 0.79 [0.76, 0.82] ^*^ |
|  | 35-49 | 0.80 [0.76, 0.85] ^*^ |
| Media exposure | No | 1 |
|  | Yes | 0.97 [0.93, 1.01] |
| Maternal occupation status | Not working | 1 |
|  | Working | 0.89 [0.86, 0.91] ^*^ |
| Marital status | Not married | 1 |
|  | Currently married | 0.89 [0.84, 0.94] ^*^ |
|  | Divorced/widowed/separated | 1.01 [0.94, 1.08] |
| Parity | 1 | 1 |
|  | 2-3 | 0.96 [0.92, 1.01] |
|  | ≥ 4 | 0.96 [0.91, 1.01] |
| Number of pregnancies | Single | 1 |
|  | Multiple | 8.49 [8.08, 8.91] ^*^ |
| Women health care decision making autonomy | Respondent alone | 1 |
|  | Jointly with partners/husband | 0.88 [0.84, 0.92] ^*^ |
|  | Husband/partner alone | 1.04 [0.99, 1.09] |
| Wanted birth | Not wanted | 1 |
|  | Wanted | 1.03 [0.97, 1.09] |
| Sex of household head | Male | 1 |
|  | Female | 0.99 [0.96, 1.03] |
| Distance to HF | Not a big problem | 1 |
|  | A big problem | 1.06 [1.03, 1.10] |
| Place of residence | Urban | 1 |
|  | Rural | 0.87 [0.83, 0.90] ^*^ |
| sub-Saharan Africa region | East Africa | 1 |
|  | Southern Africa | 1.13 [1.06, 1.20] ^*^ |
|  | Central Africa | 0.87 [0.84, 0.91] ^*^ |
|  | West Africa | 1.10 [1.06, 1.14] ^*^ |

Table 2: Multivariable multilevel binary logistic regression analysis of macrosomia in SSA

| Variable | Category | Macrosomia (Adjusted Odds Ratio (AOR) with 95% Confidence Interval (CI)) |
| --- | --- | --- |
| Maternal educational status | No | 1 |
|  | Primary | 1.26 [1.21, 1.32] ^*^ |
|  | Secondary | 1.12 [1.07, 1.18] ^*^ |
|  | Higher | 1.15 [1.05, 1.27] ^*^ |
| Household wealth status | Poorest | 1 |
|  | Poorer | 1.07 [1.02, 1.13] ^*^ |
|  | Middle | 1.06 [1.01, 1.12] ^*^ |
|  | Richer | 1.06 [1.00, 1.12] ^*^ |
|  | Richest | 1.17 [1.10, 1.24] ^*^ |
| Maternal age (in years) | 15-24 | 1 |
|  | 25-34 | 0.99 [0.95, 1.04] |
|  | 35-49 | 0.96 [0.90, 1.01] |
| Media exposure | No | 1 |
|  | Yes | 0.99 [0.96, 1.04] |
| Maternal occupation status | Not working | 1 |
|  | Working | 1.11 [1.07, 1.14] ^*^ |
| Marital status | Not married | 1 |
|  | Currently married | 1.12 [1.04, 1.21] ^*^ |
|  | Divorced/widowed/separated | 1.34 [1.23, 1.45] ^*^ |
| Parity | 1 | 1 |
|  | 2-3 | 1.25 [1.18, 1.32] ^*^ |
|  | ≥ 4 | 1.49 [1.40, 1.59] ^*^ |
| Number of pregnancies | Single | 1 |
|  | Multiple | 0.29 [0.25, 0.33] ^*^ |
| Women health care decision making autonomy | Respondent alone | 1 |
|  | Jointly with partners/husband | 0.97 [0.92, 1.01] ^*^ |
|  | Husband/partner alone | 1.01 [0.96, 1.06] |
| Wanted birth | Not wanted | 1 |
|  | Wanted | 0.79 [0.75, 0.84] ^*^ |
| Sex of household head | Male | 1 |
|  | Female | 1.02 [0.98, 1.06] |
| Distance to HF | Not a big problem | 1 |
|  | A big problem | 0.98 [0.94, 1.01] |
| Place of residence | Urban | 1 |
|  | Rural | 1.15 [1.11, 1.20] ^*^ |
| sub-Saharan Africa region | East Africa | 1 |
|  | Southern Africa | 0.69 [0.63, 0.75] ^*^ |
|  | Central Africa | 1.80 [1.73, 1.88] ^*^ |
|  | West Africa | 0.89 [0.85, 0.93] ^*^ |
